# A Statistical Exploration of QSAR Models in Cancer Risk Assessment: A Case Study on Pesticide-Active Substances and Metabolites

**DOI:** 10.3390/toxics13040299

**Published:** 2025-04-11

**Authors:** Serena Greco, Cecilia Bossa, Chiara Laura Battistelli, Alessandro Giuliani

**Affiliations:** Environment and Health Department, Istituto Superiore di Sanità, 00161 Rome, Italy; grecoserena96@gmail.com (S.G.); chiara.battistelli@iss.it (C.L.B.); alessandro.giuliani@iss.it (A.G.)

**Keywords:** (Q)SAR, non-animal approaches, computational toxicology, risk assessment, pesticides, new approach methodologies, principal component analysis

## Abstract

Data generated using new approach methodologies (NAMs), including in silico, in vitro, and in chemico approaches, are increasingly important for the hazard identification of chemicals. Among NAMs, (quantitative) structure–activity relationship (Q)SAR models occupy a peculiar position by allowing (in principle) a toxicity estimate on the sole basis of chemical structural information, leveraging upon toxicity profiles of already tested chemicals (a training set). Consequently, the metrics adopted for the estimation of both the congruence of the test chemicals with the training set and the risk categorization are of paramount importance. This paper comprises a small-scale, mainly methodological study to investigate these aspects and assess the general coherence between the results from different (Q)SAR models applied to the assessment of the carcinogenicity of pesticide-active substances and metabolites. The results of the present study underline the significant potential of using (Q)SAR models, together with limitations, such as inconsistencies in results across models and the intrinsic constraints of their applicability domain. The critical role of a priori strategies adopted in defining the applicability domain of the models is highlighted, emphasizing the need for user-transparent definitions. This is a crucial step for a sensible integration of the information coming from different NAMs.

## 1. Introduction

In traditional toxicology, animal testing has long been a widely used approach with which to assess the safety of chemicals and pharmaceuticals. In recent decades, however, there has been a growing interest in developing and adopting alternative methods to animal testing aimed at reducing, refining, and replacing [1,2] the use of animals in scientific and regulatory fields while at the same time ensuring risk evaluation at pace with the rapid increase in newly synthesized molecules.

New approach methodologies (NAMs) are any technology, methodology, approach, or combination that can provide information on chemical hazard and risk assessment to avoid the use of animal testing, including in silico, in chemico, in vitro, and ex vivo approaches [3]. They encompass a diverse range of techniques that serve as alternatives to traditional toxicity testing involving animals. These methods offer a mechanistic understanding of biologically complex endpoints. They include experimental approaches such as in vitro, in chemico methods, and in silico computational models like the (quantitative) structure–activity relationship (Q)SAR), which leverage existing data to predict chemical toxicity. Additional strategies, including grouping, read-across, and trend analysis, utilize available in vivo data to minimize the need for new animal experiments [4,5]. NAMs are faster, less expensive, and require fewer animals compared to traditional methods [5]. Integrating these methods enables a more comprehensive assessment of chemical risks, providing an ethically and scientifically valid alternative for hazard and safety evaluation. Despite their potential, NAMs cannot yet fully replace animal testing due to limitations in both their applicability and reliability.

Although alternative methods such as (Q)SAR models are foreseen in many regulatory frameworks, their acceptance by regulatory agencies to meet substance information requirements is not without its problems [6]. Enhancing these methods could support their full acceptance, further reducing reliance on animal testing [7,8]. Moreover, with the increasing use of multiple (Q)SAR models across various endpoints, attention has shifted to integrating these models for more reliable results. However, this leads to challenges, such as resolving conflicts between models and establishing robust integration strategies [9].

In the present study, the use of (Q)SAR models (already recommended for the genotoxicity assessment of pesticides and their metabolites [10]) was explored to predict the carcinogenic potential of pesticides (active substances and metabolites) from the European Food Safety Authority (EFSA) Genotoxicity Pesticides Database (https://data.europa.eu/data/datasets/database-pesticide-genotoxicity-endpoints?locale=en, accessed on 27 March 2025). Given the widely accepted link between gene mutation and tumor development, in the computational assessment of carcinogenic potential, performed using the Danish (Q)SAR software [11], we only considered the Ames (in vitro bacterial reverse mutation test) positive substances in the database. The analysis of the various endpoints of genotoxicity, mutagenicity, and carcinogenicity is beyond the scope of this paper (the reader may refer, for example, to [12] and the references therein).

This approach, which is commonly used to identify strategies for the early evaluation of carcinogenicity, ultimately minimizing negative impacts on human health and the environment while reducing or replacing animal testing [12], served as the basis for a methodological study to assess the general coherence between the results from different models.

The reliability of (Q)SAR models largely depends on the quality of the underlying chemical and biological data, which is a topic covered extensively in different reviews [13,14]. In addition to this aspect, there are various other factors to consider for the validation of (Q)SAR models and for the evaluation of the predictions derived from them [15].

Among these factors, one crucial element is related to the definition of the applicability domain (AD) and the verification of how the substance under analysis relates to it. In order to be considered as part of the AD, a target chemical should be within this space, i.e., it must be structurally similar to other chemicals used to train the model. As a consequence, even global models, which are developed to be suitable in principle for all chemical classes, might perform well only in limited portions of the chemical space. In this regard, using multiple models and integrating the generated predictions can improve the overall confidence in the results. Such integration, particularly when combined in a weight-of-evidence (WoE) approach incorporating data from in vitro and in vivo sources, supports robust toxicological assessments [16].

However, integrating the results of multiple models to provide an overall call is not an easy task. When results from independent models align, confidence in predictions increases [17]. On the other hand, highly correlated models add redundancy without improving the overall prediction quality. In practice, these aspects are difficult to investigate due to many factors at play, including the difference in algorithms, training sets, inconsistencies in reporting, etc.

The present work deals with a case study on a very limited set of chemicals aimed at assessing the state of the art of some computational models of cancer risk prediction. In this study, standard statistical techniques, such as principal component analysis, cluster analysis, and correlation analysis were applied, to evaluate concordance among different predictive models. The concordance was evaluated in terms of similarities in both predictions and the applicability domain. In particular, the elucidation of the relationship between AD overlapping and concordance of the predictions is a crucial issue in the perspective of integrating different information sources and approaches to prediction tasks.

By means of a data-driven multidimensional strategy, the present work focuses on the very first step of the integration process of different information sources (in this case, (Q)SAR approaches): their mutual superposition.

The results of the present study evidence the critical role of a priori strategies adopted for the definition of AD of the models, highlighting the need for a user-transparent definition of such strategies. The paucity of the dataset and the purely correlative approach qualify the study as a “hypothesis-generating” and not hypothesis-testing study with no ambition of sketching causative explanations.

The take-home message is that the presence of substantial test-specificity in the results signals that there is a long way to go to achieve a coherence level, enabling the routine use of these methods as stand-alone models for carcinogenicity prediction.

## 2. Materials and Methods

### 2.1. Source of Information and Data Preprocessing Phase

The initial phase of this study focused on constructing data matrices exported from The Organization for Economic Cooperation and Development (OECD) QSAR Toolbox, v4.5 (https://qsartoolbox.org/, accessed on 27 March 2025), which formed the foundation for subsequent analytical steps [18]. The OECD QSAR Toolbox software is developed by the Laboratory of Mathematical Chemistry (Burgas, Bulgaria) under the coordination of the OECD and the European Chemicals Agency (ECHA).

The OECD QSAR Toolbox allows for a reliable prediction of Ames test results, and it is widely accepted for risk estimation, notwithstanding that, in our analysis, we preferred to use the experimental Ames test results, available in the EFSA database, to select a set of potentially mutagenic substances for the analysis. All tests were performed according to the OECD test guideline n.471. The overall score for each substance was calculated according to the criteria below:

- Positive: if one strain result was positive;
- Negative: if all the strain results were negative;
- Equivocal: if some of the strain results in the same experimental study were equivocal, they could not be classified into one of the other categories.

Experimental carcinogenicity data were searched in the databases available within the OECD QSAR Toolbox, including the ISSCAN (Carcinogenicity & Mutagenicity Database from Italian National Institute of Health, ISS), ISSBIOC (Biocides and Plant Protection by ISS), ECHA (ECHA’s database), CPDB (Carcinogenic Potency Database), and ECVAM (Genotoxicity & Carcinogenicity) databases. Carcinogenicity data were available for only 75 substances (71 active substances and 6 metabolites, with 2 substances appearing in both forms). This drastically limits the numerosity of our study to only 75 statistical units.

The resulting data matrix was integrated into a single dataset and exported to an Excel spreadsheet.

A detailed summary of the selection steps is presented in Figure S1 (Supplementary Materials).

In the subsequent analyses, we focused on the subset of substances identified as Ames-positive, which consisted of 50 compounds (Table S1).

### 2.2. The Danish QSAR Software

The Danish (Q)SAR software [11] is a free online resource developed by the Technical University of Denmark (DTU) National Food Institute in cooperation and with financial support from the Danish Environmental Protection Agency (Danish EPA), the Nordic Council of Ministers and ECHA, available online since 2004 and continuously updated.

It is divided into two modules for chemical substance assessments: the Danish (Q)SAR Database and the Danish (Q)SAR Models. The Database module provides access to a comprehensive archive of model estimates from commercial, free, and DTU (Q)SAR models (in total, more than 200 models). The (Q)SAR models include endpoints for physico-chemical properties, environmental fate, bioaccumulation, eco-toxicity, absorption, metabolism, and toxicity. The database allows for the assessment of potential toxicities in chemical structures, with multiple models trained on similar datasets to predict endpoints such as genotoxicity and carcinogenicity. To enhance reliability, the database incorporates “battery calls”—majority-based predictions where at least two out of three models agree within the applicability domain.

In this study, the Danish (Q)SAR software https://qsar.food.dtu.dk/ (accessed on 27 March 2025) was employed to predict the carcinogenic potential of the selected 50 Ames-positive pesticides and primary metabolites from the EFSA Database.

Carcinogenicity predictions were available for 35 substances directly from the Database module, while the remaining 15 substances were evaluated using the Models module, yielding predictions for 13 substances.

The Database module contained carcinogenicity predictions from the following models:

- CASE Ultra (MultiCASE Inc., Beachwood, OH, USA): A system of statistical models based on fragments. The methodology decomposes the training set structures into all possible fragments between 2 and 10 heavy (non-hydrogen) atoms. The system generates simple linear chains, branched fragments, and complex substructures from combinations of multiple fragments. A structural fragment is identified as a positive alert if it resembles fragments from chemicals exhibiting toxicity for a given endpoint. Conversely, fragments are deemed non-hazardous (deactivating) if they are similar to those in chemicals deemed safe for the endpoint. Once the lists of positive and deactivating alerts are defined, CASE Ultra develops local (Q)SAR models for each alert to explain the activity of the entire training set associated with that alert. Using a stepwise regression method, local (Q)SARs are constructed based on specific molecular descriptors. The combination of positive and deactivating alerts (though not all alerts will necessarily have an associated local (Q)SAR) forms a global (Q)SAR model for a given endpoint. The results available in the Danish QSAR database refer to MultiCASE CASE Ultra v. 1.4.6.6.
- Leadscope (Leadscope Inc.; Columbus, OH, USA): Leadscope Predictive Data Miner is a software equipped with an extensive library of approximately 27,000 predefined structural features, with some derived from the training set. The software systematically analyzes chemical substructures and calculates molecular descriptors to correlate substances with their potential toxicological properties. Using statistical methods such as the χ^2^ test and Student’s *t*-test, Leadscope selects descriptors deemed most relevant for toxicological predictions. Subsequently, regression techniques, such as partial least squares regression (PLS) or partial logistic regression (PLR), are employed to construct predictive models for application to untested molecules. The results available in the Danish QSAR database refer to Leadscope Predictive Data Miner, a component of Leadscope Enterprise version 3.5.
- SciQSAR: SciQSAR software provides over 400 molecular descriptors and a variety of statistical tools to construct predictive models, including discriminant analysis models. This tool was used as implemented in the Danish (Q)SAR software [11].
- Battery: Some predictions are made using two or three independent models: CASE Ultra (CU), Leadscope Predictive Data Miner (LS), and SciQSAR (SQ). Based on the predictions of each model, a “battery prediction” is conducted using a Battery algorithm defined by the criteria reported in the DB user manual (https://qsardb.food.dtu.dk/db/index.html, accessed on 27 March 2025).

The prediction results from the models were specific to species and gender, as follows:

- Predictions from CASE Ultra (MultiCASE CASE Ultra version 1.4.) were available for the following categories:
  Male Rat, Female Rat, Rat (cumulative both sex prediction), Male Mouse, Female Mouse, Mouse (cumulative both sex prediction), Rodent (cumulative both sex and species prediction) and Liver-Specific Cancer in a Rat or Mouse.
- Predictions from Leadscope (Leadscope Enterprise version 3.5, unless otherwise specified) were available for the following categories:
  Male Rat, Female Rat, Rat (cumulative both sex prediction, Leadscope Enterprise version 3.1.1-10), Male Mouse, Female Mouse, Mouse (cumulative both sex prediction, Leadscope Enterprise version 3.1.1-10), Rodent (cumulative both sex and species prediction, Leadscope Enterprise version 3.1.1-10) and Liver-Specific Cancer in Rat or Mouse (Leadscope Enterprise version 3.1.1-10).
- Predictions from SciQSAR (SciQSAR version 3.1.00) and Battery calculation were available exclusively for Liver-Specific Cancer in a Rat or Mouse.
- Predictions performed in the Models Module of the Danish (Q)SAR Database were also limited to Liver-Specific Cancer in a Rat or Mouse.

### 2.3. Data Analysis Strategy

#### 2.3.1. Statistical Methods

To investigate the consistency of the carcinogenicity prediction models available in the Danish (Q)SAR, the following statistical techniques were applied: principal component analysis (PCA), variable clustering, and correlation analysis. These tools were utilized using SAS software (version 9.4).

PCA and clustering were applied to identify the model correlation patterns, while correlation analysis provided insight into the relationships between predictions and applicability domains.

#### 2.3.2. Principal Component Analysis

PCA is a “spectral method” that provides a parsimonious representation of a dataset spanned by n variables, projecting the data into a reduced p-dimensional (with *p* < *n*) space spanned by mutually independent p linear combinations of the original variables (principal components) and explaining the higher proportion of original variance. Dimensionality reduction is achieved by leveraging the mutual correlations among the original variables and selecting the best balance between the proportion of explained variance and the entity of reduction. This balance is made possible by the ordering of principal components and decreasing the proportion of variance explained. The correlation coefficients between the original variables and the components (loadings), in turn, allow for the extracted components to be interpreted [19].

It is noteworthy that principal component analysis is not limited to quantitative (or ordinal) variable sets but is also entirely suitable for handling binary variables, such as those related to the applicability domain [20]. Using the SAS program v.9.4, the following were calculated: (a) the principal components for the set of predictions from the carcinogenicity models in Danish (Q)SAR and (b) the principal components for the set of applicability domains of these predictions.

#### 2.3.3. Variable Clustering Analysis

Subsequently, variable clustering analysis was conducted (using the SAS VARCLUS procedure). PCA and variable clustering (oblique principal component analysis, a cognate technique of PCA) weaken the constraint of the orthogonality of components [21] and yield results complementary to PCA. The objective of clustering analysis is to group variables into clusters that are highly correlated within the group while being as independent as possible from variables in other clusters. This creates a crisp categorization of variables into groups of strongly correlated variables, which is at odds with the spectral character of PCA that generates new synthetic variables (components) in which all the variables participate with different relative contributions (loading).

This hierarchical procedure iteratively subdivides the initial set of variables until the “within-cluster correlation”/“between-cluster correlation” ratio reaches a maximum.

#### 2.3.4. Correlation Analysis

Finally, correlation analysis was conducted to examine the relationships between the model prediction variables and those related to the applicability domains. The results were defined based on the correlation coefficient, with Spearman’s coefficient selected in this case (which is essentially comparable to Pearson’s classic *r* but offers greater robustness). The correlation coefficient can range from −1 to +1. A correlation of +1 indicates a perfectly positive linear relationship between variables, whereas a correlation of −1 indicates a perfectly negative linear relationship. A correlation of 0 denotes the absence of any linear relationship.

These statistical methods provide a robust framework for evaluating the functionality and reliability of the carcinogenicity prediction models in Danish (Q)SAR. By integrating PCA, clustering, and correlation analysis, this study offers valuable insights into the predictive performance and applicability of these models.

## 3. Results and Discussion

### 3.1. Danish (Q)SAR Predictions

Since experimental carcinogenicity data were not available for most of the chemicals in the study, it was decided to perform carcinogenicity predictions using the Danish (Q)SAR software. Substances with experimental positive results in the Ames test were prioritized because such substances, if genotoxic, are also potentially carcinogenic. This analysis led to the construction of a new data matrix, which contains the results obtained through the application of the Danish (Q)SAR, as detailed below.

For most substances, the results were extracted from the Danish (Q)SAR Database module, which includes carcinogenicity predictions generated using the CASE Ultra and Leadscope models. Specifically, the available models provide predictions for the following endpoints: FDA RCA Cancer Male Rat, FDA RCA Cancer Female Rat, FDA RCA Cancer Rat, FDA RCA Cancer Male Mouse, FDA RCA Cancer Female Mouse, FDA RCA Cancer Mouse, FDA RCA Cancer Rodent, and Liver-Specific Cancer in Rat or Mouse. For the endpoint “Liver-Specific Cancer in Rat or Mouse”, additional predictions derived from the Battery and SciQSAR models were also included. For all these results, the AD status of the substance—whether it falls within (IN) or outside (OUT) the domain—was explicitly reported.

Table S2 (Supplementary Material) presents the raw output obtained using the Danish (Q)SAR models, with colors corresponding to the following specific values: POS_IN = Dark yellow; POS_OUT = Light yellow; INC_IN = Blue; INC_OUT = Light blue; NEG_IN = Dark green; and NEG_OUT = Light green.

The models were applied simultaneously to the set of substances for the defined endpoint to allow for comparison between predictions, aiming to achieve results with greater reliability (compared to predictions from a single model).

For the substances with MN_IDs 29, 91, 139, 143, 175, 177, 185, 309, 314, 366, 456, 494, 508, 628, and 695, which were not included in the software’s Database Module, the “Liver-Specific Cancer in Rat or Mouse” model from the Danish (Q)SAR Models Module was utilized. These substances appeared in the results matrix with a single prediction value, except for substances with MN_IDs 456 and 695, for which the model failed to generate predictions.

The color-coding of the cells based on their values highlights their variability in the prediction results. The lack of perfect concordance between the models reflects the complexity of predicting carcinogenicity and underscores the need for further investigation and validation.

Some of the chemicals present in the dataset fall outside the model’s AD or are deemed “inconclusive”. An “OUT of domain” classification indicates that the model might not have been designed or trained to make predictions for that specific type of substance.

The scarcity of our dataset prevents any relevant comparison between the different levels of reliability of the IN domain and OUT of domain predictions (and this is why the present work is only focused on mutual correlations between methods without any relation to their predictive power). In any case, it is worth noting that a model generating more IN domain predictions (i.e., predictions within the AD) does not automatically make it the best or most reliable model. Other considerations, such as the accuracy of predictions compared to known experimental results (when available) and additional relevant factors, must also be taken into account [7,8].

The carcinogenicity prediction results available in the Danish (Q)SAR Database for the 35 Ames-positive substances highlight discrepancies among the responses of different models. Furthermore, the analysis revealed that many predictions either fell outside the model’s applicability domain or were categorized as “inconclusive”.

### 3.2. Applicability Domain and Uncertainty Sources

**Figure 1:**
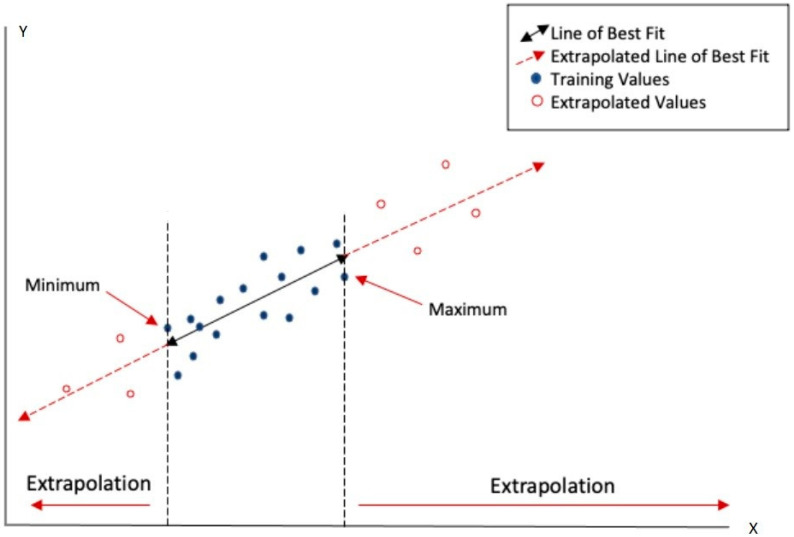
The applicability domain (AD) corresponds to the range of variation spanned by the training set within a minimum and a maximum of X (the independent variable), delimited by the dashed lines. A new statistical unit located in the AD, for which only the X value is known, acquires its estimated Y value from the best-fit equation by means of interpolation. On the contrary, a new element outside the AD can only obtain a Y estimate by means of an extrapolation that is much more affected by uncertainties.

The assessment of chemical toxicity using (Q)SAR models relies on the model’s ability to accurately predict toxicological risks based on structural and activity data from the training set. This is related to the fundamental difference between “interpolation” and “extrapolation” processes when sketched for the most basic case of bivariate regression in Figure 1.

The case of (Q)SAR models is not entirely different from the bivariate regression sketched in Figure 1: the *X* axis corresponds to a set of descriptors (e.g., chemico-physical and/or structural features) of the molecules with the width of interpolation range (AD) being estimated by suitable metrics (e.g., the distance/similarity index between the target and training set molecules). Predictions-by-interpolation are, in general, more reliable than predictions by extrapolation for the simple reason that in the case of interpolation, we do not need to make largely unjustified hypotheses on the validity of the empirical model outside the training set from where it derives. The AD determines how well the target compounds align with those used to develop the model and, thus, represent a critical factor in the reliability of predictions.

Besides the systemic exogenous errors possibly introduced by a sub-optimal AD evaluation, there are some endogenous uncertainty sources presenting a significant challenge in the chemical risk assessment by (Q)SAR approaches. The main sources of uncertainty, as reported in the literature [9], include the following:

1. Parameter uncertainty: model parameters are often calculated rather than experimentally measured, introducing unwanted (and difficult to detect) errors. Parametric variability: input variables, such as chemical descriptors, might differ depending on the software used for their calculation.
2. Structural uncertainty: each model has biases associated with the core dataset used for its development.
3. Algorithmic uncertainty: errors or numerical approximations in algorithms can affect the accuracy of results.
4. Experimental uncertainty: errors in the experimental data used to build or validate the models directly impact the reliability of (Q)SAR predictions.

### 3.3. Statistical Analysis

#### 3.3.1. Principal Component Analysis

To further investigate the functionality of the models with a particular emphasis on their mutual correlations, the carcinogenicity prediction results obtained through Danish (Q)SAR (Database and Models) were analyzed using a statistical approach based on Principal Component Analysis (PCA). The preprocessed Excel file (Table S1, in Supplementary Material) was analyzed using SAS software (version 9.4).

Table S1 was set up as follows:

- Separate columns (variables) were created for carcinogenic predictions from different models, assigning the following values: one for negative predictions, two for equivocal predictions, and three for positive predictions.
- For each column defined above, an additional variable was created to include information about the AD. Specifically, a value of 0 was assigned to OUT OF DOMAIN predictions, and a value of 1 was assigned to IN DOMAIN predictions.

Rows with missing data were removed, including those substances for which carcinogenicity predictions were made using the Models Module of the Danish (Q)SAR. This process generated a dataset made of 35 substances (statistical units).

PCA was then applied to the above dataset, in which the 35 chemicals are defined by 36 variables (18 for predictions and 18 for ADs). Principal components were extracted by two independent procedures for prediction and AD spaces. The cutoff for selecting signal components was set at eigenvalue = 1, following the “Kaiser criterion” [18]. Both datasets gave rise to a six-component solution (Table 1 and Table 2).

The comparison of Table 1 and Table 2 points to an almost coincident profile of explained variance and, thus, to a similar complexity (the more complex a system is, the higher the number of components necessary to explain a consistent proportion of variance) of the prediction and AD spaces. The need for six independent axes to accommodate the statistical units initially defined in an 18-dimension space corresponds to a relatively weak correlation structure in the system studied in both the prediction and ADs, suggesting the presence of local- and model-specific latent rules. The absence of general unifying rules across the different models will be confirmed by subsequent analyses.

Table 3 and Table 4 report the component loading profiles (factor pattern) of prediction (test) and AD spaces. The component loadings are the Pearson correlation coefficient between the original variables and components: the variables most relevant for assigning a meaning to a given component are those with higher (absolute values) loadings. Analogously, if a component shows an elevated (absolute) loading value for a given variable, it can be inferred that this component is particularly representative of that variable. This can reflect differences among the models or capture different aspects of the data highlighted by the principal components. The presence of particular loading profiles suggests a distinctive correlation structure in the dataset. The “leading” variables for each component are highlighted in Table 3 and Table 4. The adopted criterion for considering a variable relevant for component interpretation was linked to both the actual loading value and the variance explained by each variable; consequently, minor components can derive their meaning using less correlated (lower loading) variables with respect to major components endowed with a greater eigenvalue (and thus explaining the higher proportion of the general correlation structure).

In this case, the components mirror independent constellations of latent classification rules, some of which are shared (different models with relevant loadings on the same component) and some of which are idiosyncratic (only one model has relevant loading on a given component).

As shown by the highlighted values of Table 3, PCtest1 is predominantly influenced by predictions from the Leadscope models, especially those corresponding to a Male Rat, Male Mouse, Rat, Mouse, and Female Rat. This result suggests a substantial consistency in the Leadscope system across rodent species and genders.

The second principal component has predictions (in descending order) from SciQSAR as the leading variable for Liver-Specific Cancer in a Rat or Mouse, CASE Ultra for Rodents, Battery for Liver-Specific Cancer in a Rat or Mouse, and Leadscope for Liver-Specific Cancer in a Rat or Mouse.

The third principal component mainly correlates with predictions from CASE Ultra (in descending order) for the Female Mouse, Mouse, and Male Mouse. From this observation, it can be inferred that CASE Ultra predictions for mice species exhibit a distinctive trend that is not correlated with other predictions and is characterized by more consistent results in mice species.

From Table 3, it is evident that different models exhibit distinct patterns of correlation with the principal components. Some Leadscope predictions are strongly correlated with PCtest1; certain liver-specific predictions and one CASE Ultra prediction are strongly associated with PCtest2; and CASE Ultra predictions for mice species are closely tied to PCtest3.

Given that minor components are progressively more affected by noise, the description can be reasonably limited to the first three principal components. These major components outline a clear relational structure: the relative independence of Leadscope and CASE Ultra systems (associated with different components) and the uniqueness of liver-specific cancer in rodents.

This last finding represents an important confirmation of the biological plausibility of the PCA results, which spontaneously highlights a well-known phenomenon in toxicology: the peculiarity of rodent liver tumors, which develop through a specific mechanism (peroxisome proliferation) [22] distinct from all other types of tumors.

The main “take-home” messages originating from the PCA of prediction space are as follows:

- The relative independence of the predictions from Leadscope and CASE Ultra systems (associated with different components).
- The uniqueness of Liver-Specific Cancer in a Rat or Mouse.

Table 4 details the factor pattern for the ADs of the models. These patterns stem from the binary variables defining the pertinence of the predictions to the applicability domain of different models. Here, it is worth remembering that binary variables are appropriate for principal component analysis, as evidenced by Cox [20].

The main features emerging from the AD space factor pattern are as follows:

- PCdom1 exhibits strong positive correlations with R_CASE_DOM1, R_CASE_DOM, RF_CASE_DOM, RM_CASE_DOM, FM_CASE_DOM, LS_R_M_BATT_DOM, LS_R_M_CASE_DOM, and M_CASE_DOM. The strong correlation of R_CASE_DOM1 with PCdom1 (0.791) suggests that this AD is particularly influential in shaping PCdom1.
- PCdom1 also shows a strong negative correlation with MM_LEAD_DOM, indicating that the ADs of the two models are complementary. Substances falling within the domain of one model are generally outside the domain of the other. This observation raises a very important point for model integration, which we will discuss in the following.
- PCdom2 is primarily associated with R_LEAD_DOM, MM_CASE_DOM, RF_LEAD_DOM, and M_LEAD_DOM.
- PCdom3 has strong positive correlations with the applicability domains of RM_LEAD_DOM and R_LEAD_DOM1.

Focusing on the first component, i.e., the main order parameter shaping the relational structure of the AD and, consequently, the best proxy of the overall structure of the AD shared by all the considered models, we can derive the following consequences:

1. R_CASE_DOM1 has a very strong correlation with PCdom1 (0.791), making this component highly representative of the AD of CASE Ultra for Rodent (Rodent) that, in turn, can be considered to occupy a central position among the different (Q)SAR models.
2. RF_CASE_DOM and RM_CASE_DOM (the ADs of CASE Ultra for a Female Rat and Male Rat) also show strong positive correlations with PCdom1 (0.618 and 0.614, respectively). This highlights the substantial internal coherence of CASE Ultra for the Rodent, Female Rat, and Male Rat, distinguishing them significantly from the ADs defining subsequent components (PCdom2 and PCdom3).
3. MM_LEAD_DOM has a significant negative correlation (−0.587) with PCdom1. This implies that when MM_LEAD_DOM increases, the value of PCdom1 decreases, confirming the complementarity of ADs of CASE Ultra and Leadscope that can be leveraged for the integration of the two methods.
4. Most other variables exhibit moderate positive correlations with PCdom1, indicating some association but one that is not as strong as the variables mentioned above.

While some ADs strongly correlate with a single principal component, others are associated with multiple components. This could reflect the overlap in ADs for some predictions. These distinct correlations underscore the importance of considering each domain both individually and in combination when interpreting the overall results of the analysis.

In this case, the uniqueness of liver-specific predictions does not emerge because the domain only reflects the inclusion or exclusion of certain chemicals in the training set of the predictive model, as this is unrelated to the biological effects of these substances (which form the basis of the PCA performed on the “test” variables).

The most puzzling result emerging from the analysis is the opposite sign of the loading pattern of different models: this could be in light of the fact that some models were “trained” on largely distinct sets of substances. This, in turn, may explain (alongside the inherent uncertainty in carcinogenicity experiments) the low overlap of their predictions. Moreover, the use of different metrics for deciding the alignment of a target chemical with the AD can also contribute to the largely non-overlapping applicability features.

The paucity of the analyzed dataset prevents a more in-depth analysis of the determinants of the observed correlation pattern, but, as we stressed before, this is the main methodological exercise with which to demonstrate the usefulness of a data-driven multidimensional approach to optimize the efficacy of test batteries. What is for sure is that a strong dependence on specific models and their peculiarities tells us that we are still far from achieving a generalizable predictive model for carcinogenicity.

The issue of the model’s idiosyncratic properties will emerge in a still more evident way in the following analyses.

#### 3.3.2. Variable Clustering Analysis

**Figure 2:**
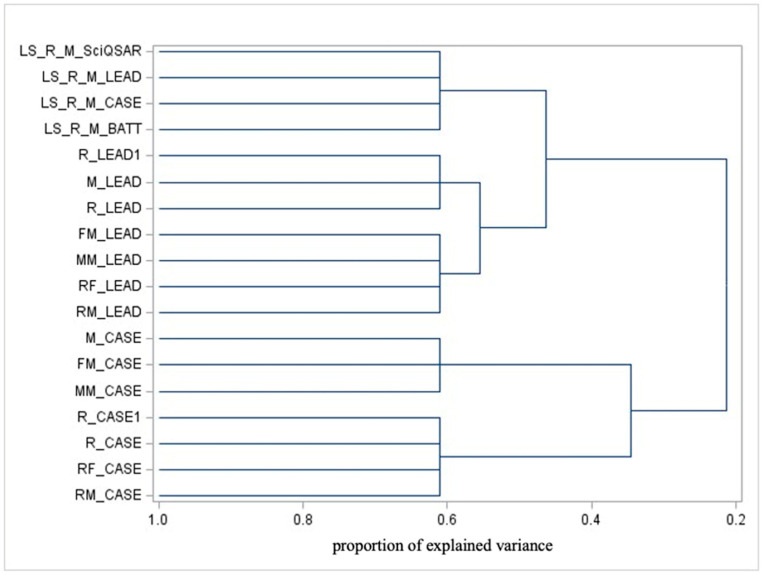
Dendrogram illustrating the cluster analysis applied to model predictions.

**Figure 3:**
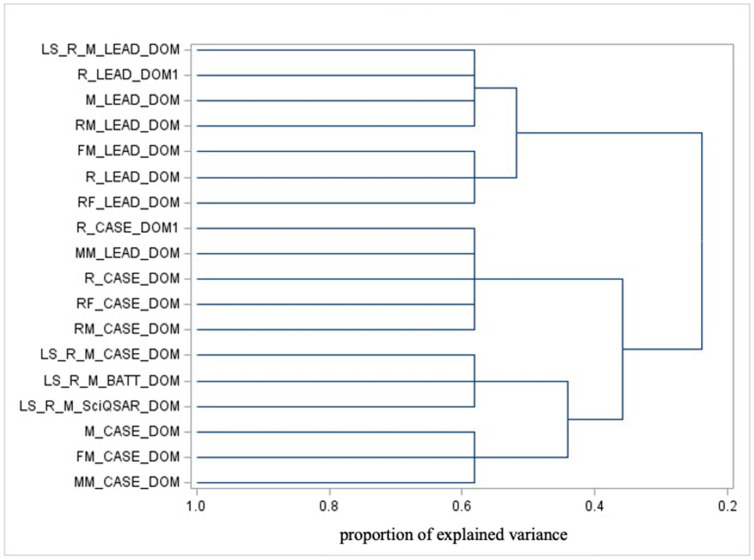
Dendrogram illustrating the cluster analysis of ADs.

Combining PCA with cluster analysis can help identify hidden patterns or structures in the data that may not be evident when using either technique alone. This section presents the results obtained from applying cluster analysis to variables. Figure 2 and Figure 3 show the dendrograms for test variables and domain variables, respectively. As we stress above, as a spectral method, PCA is useful to identify the latent rules and configurations at the basis of the dataset, while variable clustering focuses on the classification of models based on their similarities in both prediction and AD profiles.

Observing the dendrogram in Figure 2, which focuses on model predictions, the most significant separation occurs (from right to left, which is the ordering correspondent for progressively finer detail and, thus, increasingly small clusters) between the following:

1. CASE Ultra predictions, which are further clustered as follows:
  - Mouse, Female Mouse, and Male Mouse.
  - Rodent, Rat, Female Rat, and Male Rat.
2. Predictions from various models (SciQSAR, Leadscope, CASE Ultra, Battery) targeting liver-specific endpoints (a Rat or Mouse).
3. Leadscope predictions are further grouped as follows:
  - Rodent, Mouse, and Rat.
  - Female Mouse, Male Mouse, Female Rat, and Male Rat.

From this clustering, the following key insights emerge:

1. Predictions from CASE Ultra tend to cluster by species, suggesting that this model may incorporate species-specific biological features. Within species-based clusters, additional finer distinctions appear based on sex.
2. Predictions for liver-specific endpoints are clearly separated from other variables (notably, they are not differentiated by sex or gender).
3. Predictions from the Leadscope cluster differ from CASE Ultra, suggesting differences in how these models prioritize or interpret variables such as species and sex.

CASE Ultra’s cluster location shows that individuals of the same species but different sexes are grouped together; this implies that, within the context of the data analyzed, sex-related differences are less significant drivers of variability compared to species-specific features. This highlights species similarity outweighing sex-based variation.

This does not imply that sex is irrelevant to toxicity or carcinogenicity. Biological sex can influence the metabolism, distribution, and response to chemicals, with potential impacts manifesting in processes like biotransformation rates, protein binding, and immune responses, but species (as expected) is a more relevant source of diversity.

The distinct separation between CASE Ultra and Leadscope predictions reflects their differing methodologies or mechanisms, with potential explanations including the following:

1. Distinct Algorithms or Methodologies: The models may utilize different computational approaches, leading to divergent outputs.
2. Diverse Training Datasets: CASE Ultra and Leadscope might rely on different datasets or inclusion criteria that influence the predictions.
3. Different Objectives: Despite both focusing on carcinogenicity, the models may prioritize specific chemical classes or mechanisms differently.
4. Accuracy and Reliability: One model may demonstrate greater consistency or reliability, leading to clearer clustering distinctions.

The clear separation between predictions suggests that considering both models together provides complementary insights, allowing for a more robust evaluation of a substance’s carcinogenic potential.

#### 3.3.3. Domain Clustering

The cluster analysis of AD highlights the similarities between chemical spaces where predictions are deemed reliable based on the training dataset. Figure 3 depicts the dendrogram for domain variables.

From Figure 3, focusing on ADs, the main separations (from right to left) include the following:

1. CASE Ultra’s applicability domain vs. liver-specific domains, which can be further divided into the following:
  Specific instances of CASE Ultra and liver-specific domains (except for liver-specific Leadscope, which clusters with general Leadscope AD).
2. Leadscope’s AD.

The key interpretations are as follows:

- The distinction between CASE Ultra and Liver-Specific domains reflects differences in their focus or training sets. Liver-specific models likely emphasize specific mechanisms and subsets of substances.
- The separation of Leadscope’s domain from the others underscores its unique training set, focus, or methodology.
- Leadscope’s liver-specific domain clustering with the general Leadscope domain may indicate shared foundational characteristics coming from identical model specifications.

It is worth remembering that the classification of applicability domains cannot reflect, at odds with the test variable clustering, any biological feature for the simple reason that the alignment of a chemical in a specific AD happens independently of the specific test results.

#### 3.3.4. Correlation Analysis

The correlation analysis investigates relationships between model predictions and their applicability domains, using Spearman’s correlation coefficient for its robustness against outliers [23]. Table 5 shows the correlation matrix between the test and domain variables (such as correlation coefficients and *p*-values), highlighting the correlation between the test and AD within the same model.

Key Insights:

1. RM_CASE_DOM vs. RM_CASE: Strong negative correlation (−0.763, *p* < 0.0001), suggesting that as RM_CASE increases, RM_CASE_DOM decreases (or vice versa).
2. RM_LEAD_DOM vs. RM_LEAD: Moderate negative correlation (−0.471, *p* = 0.0043).
3. RF_CASE_DOM vs. RF_CASE: Strong negative correlation (−0.605, *p* = 0.0001).
4. RF_LEAD_DOM vs. RF_LEAD: Moderate negative correlation (−0.480, *p* = 0.0035).
5. R_CASE_DOM vs. R_CASE: Strong negative correlation (−0.746, *p* < 0.0001).

Other correlations were relatively weak and not statistically significant (*p* > 0.05).

Correlations between the test predictions and their respective ADs were consistently negative, suggesting that substances classified as “OUT of domain” tend to be labeled as positive. This indicates a conservative tendency in the models, where maximum uncertainty (OUT of domain) often results in a positive classification.

However, this conservatism might lead to a higher incidence of false positives, emphasizing the importance of interpreting predictions within the broader context of experimental data and risk assessment.

The exploratory characteristic of the analysis made it almost mandatory to scatter the findings, important information, and take-home messages across the different sections. While this style could be detrimental to clarity, it is a direct consequence of the nature of the hypothesis generation of the adopted strategy of analysis [19], which creates a collapse between the results and their interpretation. In any case, this pilot study addresses some issues of in silico methods for carcinogenesis prediction that should still be considered “another piece of information”, generating the largely tacit knowledge of experts in the field. It is worth noting that Danish QSAR is not the only tool to face carcinogenicity estimation; instead, we chose it for its public, free-of-charge character and its consequent widespread use in the toxicologist community. Moreover, the paucity of the chemicals with a full range profile of both genotoxicity and carcinogenicity tests did not allow for the further insertion of layers of analysis corresponding to among-tools comparisons, different mechanisms of action, and other important factors like the route of exposure or parent vs. metabolism prediction.

## 4. Conclusions

The findings of this study clearly highlight the challenges and opportunities associated with using (Q)SAR models in the current context. While these tools have shown significant potential, certain limitations persist, such as inconsistencies in the results across models and the intrinsic constraints of their ADs. Although considering both models together would provide complementary insights, allowing for a more robust evaluation of a substance’s carcinogenic potential, the issues highlighted the need for further refinement and optimization to enhance their reliability and predictivity.

(Q)SAR models were originally developed in medicinal chemistry, which is a very different context with respect to toxicity predictions. While medicinal chemistry deals with a homologous series of chemicals [24] sharing a common mechanism of action, toxicity prediction faces diverse chemical moieties.

Achieving the comprehensive sampling of the chemical universe so as to replace the original mechanistic-based applicability with “statistical applicability” remains a critical step but is still far from being realized.

ML techniques represent a promising development in advancing (Q)SAR models toward this goal. According to recent studies [25], ML has the potential to revolutionize scientific processes by automating pattern extraction and enhancing predictive accuracy.

It is worth noting that the advancements driven by ML are not a pure consequence of a more powerful computational technique. As a matter of fact, there is no truly “theory-free” approach in ML [25,26]; each step in the process—from selecting descriptors to defining datasets—involves theoretical choices that exert a major influence on the outcomes. The success of integrating ML depends heavily on the quality of input data and the preliminary optimization of datasets used to train these systems.

In this “pilot study”, which aimed to describe the state of the art of regularly used in silico methods in cancer risk prediction, we highlighted that the convergence of predictive methods is still weak toward a shared optimum, as evidenced by the model specificity of both predictions and ADs. The take-home message is the urgent need to bridge the gap between computational and theoretical views of toxicity predictions. Relational approaches, such as Physics-Informed Neural Networks (PINNs) [27], provide a promising path forward, offering a balance between accuracy and interpretability.

The consistency between toxicological knowledge (e.g., rodent liver cancer peculiarities) and emerging between-test similarities is a confirmation of the usefulness of exploratory statistical analysis of the prediction models. Moreover, the results highlight the need to increase the transparency of the rules adopted in the alignment of a target molecule to the AD of different models so that toxicologists can increase their awareness of the automatic prediction procedure to produce a more sensible risk assessment.

In conclusion, the integration of ML techniques with (Q)SAR models holds significant promise. By addressing current limitations, such as domain applicability and inconsistencies, and combining computational advances with theoretical rigor, these approaches could revolutionize the evaluation of chemical risks. This integration would not only improve the quality of predictive analyses but also have profound ethical and scientific implications, paving the way for a more innovative and sustainable future in toxicology.

## Figures and Tables

**Table 1:** Explained variance from Principal Component Analysis (PCA) applied to predictions for the 35 substances. The cutoff for defining extracted components (signal) was the eigenvalue = 1 (Kaiser’s criterion).

PCtest (Predictions)	Eigenvalue	Cumulative Variance Explained
PCtest1	3.833	0.213
PCtest2	3.037	0.382
PCtest3	2.568	0.524
PCtest4	1.740	0.621
PCtest5	1.165	0.686
PCtest6	1.070	0.745

**Table 2:** Principal components for applicability domain (AD) variables across various models.

PCdom (Domain)	Eigenvalue	Cumulative Variance Explained
PCdom1	4.294	0.239
PCdom2	2.782	0.393
PCdom3	1.946	0.501
PCdom4	1.751	0.598
PCdom5	1.281	0.670
PCdom6	1.088	0.730

**Table 3:** The loading pattern for the model predictions. Values in bold indicate particularly strong correlations between the original variables and the principal components, making them the most relevant for assigning meaning to the components. A threshold of r > |0.50| was chosen considering both the dataset numerosity and the variance explained by the components; italics point to sub-threshold values that carry some information for variable/component interpretation.

	PCtest1	PCtest2	PCtest3	PCtest4	PCtest5	PCtest6
RM_CASE	0.440	0.353	−0.355	−0.133	0.401	−0.153
RM_LEAD	**0.747**	−0.185	−0.0433	0.220	−0.104	0.111
RF_CASE	*0.449*	0.357	−0.328	**−0.542**	−0.128	0.239
RF_LEAD	*0.493*	−0.0318	−0.227	*0.483*	0.320	0.313
R_CASE	0.436	0.351	−0.395	**−0.602**	0.110	0.133
R_LEAD	**0.625**	−0.130	−0.235	0.316	−0.140	−0.0583
MM_CASE	0.268	0.388	**0.564**	0.393	−0.0574	0.171
MM_LEAD	**0.712**	−0.200	−0.0172	0.114	0.111	−0.029
FM_CASE	0.168	0.345	**0.865**	−0.0778	0.0672	−0.0759
FM_LEAD	0.450	−0.364	0.172	0.208	0.295	0.343
M_CASE	0.160	0.316	**0.837**	−0.236	0.243	−0.0777
M_LEAD	**0.608**	0.0707	0.146	−0.276	−0.421	0.081
R_CASE1	0.480	**0.690**	0.0973	−0.0705	0.107	−0.0975
R_LEAD1	0.551	0.119	−0.0276	0.241	**−0.591**	−0.362
LS_R_M_BATT	−0.385	**0.634**	−0.383	0.224	−0.0104	0.139
LS_R_M_CASE	0.0942	*0.467*	−0.270	0.295	0.286	*−0.493*
LS_R_M_LEAD	−0.310	**0.610**	0.0271	0.192	−0.216	**0.535**
LS_R_M_SciQSAR	−0.250	**0.761**	−0.144	0.315	−0.104	−0.0414

**Table 4:** Factor pattern for the ADs of the models. Values in bold indicate particularly strong correlations between the original variables and the principal components. A threshold of r > |0.50| was chosen considering both the dataset numerosity and the variance explained by the components.

	PCdom1	PCdom2	PCdom3	PCdom4	PCdom5	PCdom6
LS_R_M_SciQSAR_DOM	0.348	0.412	0.049	0.476	0.139	−0.171
RM_CASE_DOM	**0.614**	−0.0263	0.277	−0.141	**0.501**	0.322
RM_LEAD_DOM	−0.187	0.459	**0.694**	0.129	−0.127	0.189
RF_CASE_DOM	**0.618**	0.0727	0.0965	0.165	**−0.499**	0.227
RF_LEAD_DOM	−0.354	**0.541**	−0.419	0.328	−0.282	0.180
R_CASE_DOM	**0.703**	−0.174	0.254	0.0277	−0.00828	0.349
R_LEAD_DOM	−0.304	**0.751**	0.0338	0.0134	−0.00811	0.0689
MM_CASE_DOM	0.444	**0.588**	−0.114	−0.320	0.0916	−0.0158
MM_LEAD_DOM	**−0.587**	0.257	−0.208	−0.052	0.291	−0.290
FM_CASE_DOM	**0.565**	0.390	**−0.511**	−0.223	0.0700	0.0804
FM_LEAD_DOM	−0.379	0.430	−0.198	0.125	0.312	**0.573**
M_CASE_DOM	**0.535**	0.152	−0.444	−0.483	0.131	−0.142
M_LEAD_DOM	−0.164	**0.508**	0.0331	**−0.531**	−0.399	−0.00694
R_CASE_DOM1	**0.791**	−0.114	−0.115	−0.0862	0.00396	0.046
R_LEAD_DOM1	−0.122	0.433	**0.533**	−0.190	0.398	−0.0998
LS_R_M_BATT_DOM	**0.556**	0.301	0.179	0.446	0.163	−0.442
LS_R_M_CASE_DOM	**0.505**	0.261	−0.293	**0.594**	−0.0268	−0.0583
LS_R_M_LEAD_DOM	0.383	0.347	0.406	−0.267	−0.330	−0.245

**Table 5:** Correlation matrix between the test and domain variables. Each cell contains two values: (1) The Pearson correlation coefficient. (2) The *p*-value, indicating statistical significance. Correlations between the test and AD within the same model are shown in bold.

	RM_CASE	RM_LEAD	RF_CASE	RF_LEAD	R_CASE
RM_CASE_DOM	**−0.763** <0.0001	0.022 0.8986	0.041 0.8144	−0.0489 0.7843	−0.313 0.0669
RM_LEAD_DOM	0.103 0.5555	**−0.471** 0.0043	0.042 0.8088	0.099 0.5717	0.081 0.6447
RF_CASE_DOM	−0.043 0.8078	0.100 0.5654	**−0.604** 0.0001	0.00000 1.0000	−0.4011 0.0169
RF_LEAD_DOM	0.272 0.1140	−0.415 0.0131	−0.041 0.8144	**−0.480** 0.0035	0.207 0.2333
R_CASE_DOM	−0.480 0.0035	0.270 0.1167	−0.339 0.0461	0.148 0.3948	**−0.746** <0.0001

## Data Availability

The data used in this study are publicly available in the sources cited in the Methods section. The processed dataset used for statistical calculations is available in the Supplementary Material (Table S1.xlsx).

## Supplementary Materials

The following supporting information can be downloaded at https://www.mdpi.com/article/10.3390/toxics13040299/s1. Table S1. A portion of the Excel file prepared for SAS processing and PCA application. Abbreviations used for species: RM = Male Rat; RF = Female Rat; R = Rat; MM = Male Mouse; MF = Female Mouse; M = Mouse; R_1 = Rodent; and LS = Liver Specific. Table S2. Raw output obtained using the Danish (Q)SAR software (Database Module and Models Module for substances not included in the Database Module), where POS_IN = positive prediction within the applicability domain; POS_OUT = positive prediction outside the applicability domain; INC_IN = inconclusive prediction within the applicability domain; INC_OUT = inconclusive prediction outside the applicability domain; NEG_IN = negative prediction within the applicability domain; NEG_OUT = negative prediction outside the applicability domain. Data source: “Danish (Q)SAR Database, Division of Diet, Disease Prevention and Toxicology, National Food Institute, Technical University of Denmark. Food Institute, Technical University of Denmark [11]. Figure S1. A comprehensive summary of the steps performed on the EFSA Genotoxicity database. It includes counts and classifications of active substances (ASs) and metabolites (METs), along with the application of results of the Ames test and experimental carcinogenicity data, categorized as POS (positive), NEG (negative), EQ (equivocal), and ND (no data available). The data are visually represented using a color-coded schema: Green: genotoxicity data; Yellow: carcinogenicity data; Pink: statistical analysis; Orange: software used in different steps; and Blue: source database and any modifications applied. It is important to note that some substances appear both as active substances and metabolites, but they are counted individually in the overall totals.

## Abbreviations

The following abbreviations are used in this manuscript:

AD	Applicability domain
ASs	Active substances
CPDB	Carcinogenic Potency Database
CU	CASE Ultra
Danish EPA	Danish Environmental Protection Agency
DTU	Technical University of Denmark
ECHA	European Chemicals Agency
ECVAM	Genotoxicity & Carcinogenicity Database by the European Reference Laboratory for Alternatives to Animal Testing
EFSA	European Food Safety Authority
EQ	Equivocal
IN	In the domain
ISS	Italian National Institute of Health
ISSBIOC	Biocides and Plant Protection Database by ISS
ISSCAN	Carcinogenicity & Mutagenicity Database by ISS
LS	Leadscope Predictive Data Miner
MET	Metabolites
ML	Machine learning
MM/MF	Mouse Male/Mouse Female
MN_ID	Unique identifier of substances under analysis
NAMs	New approach methodologies
ND	No data available
NEG	Negative result
OECD	Organisation for Economic Cooperation and Development
OUT	Out of the domain
PCA	Principal Component Analysis
PINNs	Physics-Informed Neural Networks
PLR	Partial logistic regression
PLS	Partial least squares regression
POS	Positive result
RM/RF	Rat Male/Rat Female
(Q)SAR	(Quantitative) structure–activity relationship
SQ	SciQSAR
WoE	Weight of evidence

## References

[1] Tannenbaum J., Bennett B.T. (2015). Russell and Burch’s 3Rs then and now: The need for clarity in definition and purpose. J. Am. Assoc. Lab. Anim. Sci..

[2] Jean-Quartier C., Jeanquartier F., Jurisica I., Holzinger A. (2018). In silico cancer research towards 3R. BMC Cancer.

[3] ECHA New Approach Methodologies in Regulatory Science Proceedings of a Scientific Workshop. Helsinki, April 2016. https://echa.europa.eu/documents/10162/21838212/scientific_ws_proceedings_en.pdf/a2087434-0407-4705-9057-95d9c2c2cc57.

[4] ECHA, Non-Animal Approaches Current Status of Regulatory Applicability Under The REACH, CLP and Biocidal Products Regulations. https://echa.europa.eu/documents/10162/22931011/non_animal_approcches_en.pdf/87ebb68f-2038-f597-fc33-f4003e9e7d7d.

[5] ECHA (2023). The Use of Alternatives to Testing on Animals for the REACH Regulation. https://echa.europa.eu/documents/10162/23919267/230530_117_3_alternatives_test_animals_2023_en.pdf/9cfc291e-9baf-ffa2-466c-2bc2c6f06b8e?t=1685428213290.

[6] Macmillan D.S., Bergqvist A., Burgess-Allen E., Callan I., Dawick J., Carrick B., Ellis G., Ferro R., Goyak K., Smulders C. (2024). The last resort requirement under REACH: From principle to practice. Regul. Toxicol. Pharmacol..

[7] Gissi A., Tcheremenskaia O., Bossa C., Battistelli C.L., Browne P. (2024). The OECD (Q)SAR Assessment Framework: A tool for increasing regulatory uptake of computational approaches. Comput. Toxicol..

[8] OECD (2024). (Q)SAR Assessment Framework: Guidance for the Regulatory Assessment of (Quantitative) Structure Activity Relationship Models and Predictions, Second Edition. Series on Testing and Assessment No. 405. https://www.oecd.org/en/publications/q-sar-assessment-framework-guidance-for-the-regulatory-assessment-of-quantitative-structure-activity-relationship-models-and-predictions-second-edition_bbdac345-en.html.

[9] Benfenati E., Chaudhry Q., Gini G., Dorne J.L. (2019). Integrating in silico models and read-across methods for predicting toxicity of chemicals: A step-wise strategy. Environ. Int..

[10] EFSA PPR Panel (2016). Guidance on the establishment of the residue definition for dietary risk assessment EFSA Panel on Plant Protection Products and their Residues (PPR). EFSA J..

[11] Technical University of Denmark Danish (Q)SAR database/(Q)SAR Models. Division of Diet, Disease Prevention and Toxicology, National Food Institute, Technical University of Denmark. https://qsar.food.dtu.dk/.

[12] Bossa C., Benigni R., Tcheremenskaia O., Battistelli C.L. (2018). (Q)SAR methods for predicting genotoxicity and carcinogenicity: Scientific rationale and regulatory frameworks. Methods in Molecular Biology.

[13] Battistelli C.L., Bossa C. (2025). Development, Use, and Validation of (Q)SARs for Predicting Genotoxicity and Carcinogenicity: Experiences from Italian National Institute of Health Activities. Computational Toxicology: Methods and Protocols.

[14] Madden J.C., Enoch S.J., Paini A., Cronin M.T.D. (2020). A Review of In Silico Tools as Alternatives to Animal Testing: Principles, Resources and Applications. Altern. Lab. Anim..

[15] OECD (2004). OECD Principles for the Validation for Regulatory Purposes of (Quantitative) Structure-Activity Relationship Models. https://www.oecd.org/content/dam/oecd/en/topics/policy-sub-issues/assessment-of-chemicals/oecd-principles-for-the-validation-for-regulatory-purposes-of-quantitative-structure-activity-relationship-models.pdf.

[16] Hardy A., Benford D., Halldorsson T., Jeger M.J., Knutsen H.K., More S., Naegeli H., Noteborn H., Ockleford C., Ricci A. (2017). Guidance on the use of the weight of evidence approach in scientific assessments. EFSA J..

[17] ICH M7(R2) ICH M7(R2) Guideline on Assessment and Control of DNA Reactive (Mutagenic) Impurities in Pharmaceuticals to Limit Potential Carcinogenic Risk Step 5 July 2023. https://www.ema.europa.eu/en/documents/scientific-guideline/ich-m7r2-guideline-assessment-and-control-dna-reactive-mutagenic-impurities-pharmaceuticals-limit-potential-carcinogenic-risk-step-5_en.pdf.

[18] Benigni R., Battistelli C.L., Bossa C., Giuliani A., Fioravanzo E., Bassan A., Fuart Gatnik M., Rathman J., Yang C., Tcheremenskaia O. (2019). Evaluation of the applicability of existing (Q)SAR models for predicting the genotoxicity of pesticides and similarity analysis related with genotoxicity of pesticides for facilitating of grouping and read across. EFSA Support. Publ..

[19] Giuliani A. (2017). The application of principal component analysis to drug discovery and biomedical data. Drug Discov. Today.

[20] Cox D.R. (1972). The Analysis of Multivariate Binary Data. Appl. Stat..

[21] Richman M.B. (1986). Rotation of principal components. J. Climatol..

[22] Corton J.C., Peters J.M., Klaunig J.E. (2019). The PPARα-dependent rodent liver tumor response is not relevant to humans: Addressing misconceptions. Arch. Toxicol..

[23] de Winter J.C.F., Gosling S.D., Potter J. (2016). Comparing the Pearson and Spearman correlation coefficients across distributions and sample sizes: A tutorial using simulations and empirical data. Psychol. Methods.

[24] Hansch C. (1969). Quantitative approach to biochemical structure-activity relationships. Acc. Chem. Res..

[25] The Royal Society (2024). Science in the Age of AI: How Artificial Intelligence is Changing the Nature and Method of Scientific Research. https://royalsociety.org/-/media/policy/projects/science-in-the-age-of-ai/science-in-the-age-of-ai-report.pdf.

[26] Giuliani A. (2024). System Science Can Relax the Tension Between Data and Theory. Systems.

[27] Cai S., Mao Z., Wang Z., Yin M., Karniadakis G.E. (2021). Physics-informed neural networks (PINNs) for fluid mechanics: A review. Acta Mech. Sin..

