# Supplementary material for: A Statistical Exploration of QSAR Models in Cancer Risk Assessment: A Case Study on Pesticide-Active Substances and Metabolites
Source: Toxics. 2025 Apr 11;13(4):299. doi: 10.3390/toxics13040299 (PMC12030765; doi:10.3390/toxics13040299)
Supplement: Supplementary file 1 [file toxics-13-00299-s001.zip › Supplementary Materials.pdf]

Supplementary Materials

Table *S*1. (excel file Table S1.xlsx) A portion of the Excel file prepared for SAS processing and PCA application. Abbreviations used for species: RM= Male Rat; RF=Female Rat; R=Rat; MM=Male Mouse; MF=Female Mouse; M=Mouse; R_1=Rodent; and LS=Liver Specific.

Table S2 illustrates the results of predictions performed using Danish (Q)SAR software.

Table *S*2: The raw output obtained using the Danish (Q)SAR software (the Database Module and, alternatively, Models Module for substances not included in the Database Module), where POS_IN = positive prediction within the applicability domain; POS_OUT = positive prediction outside the applicability domain; INC_IN = inconclusive prediction within the applicability domain; INC_OUT = inconclusive prediction outside the applicability domain; NEG_IN = negative prediction within the applicability domain; and NEG_OUT = negative prediction outside the applicability domain. Data source: Danish (Q)SAR Database, Division of Diet, Disease Prevention and Toxicology, National Food Institute, Technical University of Denmark. Food Institute, Technical University of Denmark, [11].

	A	B	C	D	E	F	G	H	I	J	K	L	M	N	O	P	Q	R	S	T	U	V
1	MN_	ID_Pc	Ove	FDA RCA Cancer Male Rat		FDA RCA Cancer Female Rat		FDA RCA Cancer Rat		FDA RCA Cancer Male Mouse		FDA RCA Cancer Female Mouse		FDA RCA Cancer Mouse		FDA RCA Cancer Rodent		Liver Specific Cancer in Rat or Mouse				Models (Live
2				CASE Ultra	Leadscope	CASE Ultra	Leadscope	CASE Ultra	Leadscope	CASE Ultra	Leadscope	CASE Ultra	Leadscope	CASE Ultra	Leadscope	CASE Ultra	Leadscope	Battery	CASE Ultra	Leadscope	SciQSAR	
3	4	1		POS_OUT	INC_OUT	NEG_OUT	INC_OUT	INC_OUT	INC_OUT	INC_OUT	NEG_IN	INC_OUT	INC_OUT	INC_OUT	NEG_OUT	NEG_OUT	NEG_OUT	INC_OUT	INC_OUT	NEG_OUT	NEG_OUT	
4	16	2	3	POS_IN	POS_IN	POS_IN	INC_OUT	POS_IN	POS_IN	POS_IN	INC_OUT	POS_IN	POS_OUT	POS_IN	POS_IN	POS_IN	POS_IN	NEG_IN	NEG_OUT	NEG_IN	NEG_IN	
5	17	3	1	POS_OUT	NEG_IN	NEG_IN	NEG_IN	POS_OUT	NEG_IN	NEG_IN	NEG_OUT	POS_IN	NEG_IN	POS_OUT	INC_OUT	NEG_IN	NEG_OUT	NEG_IN	NEG_IN	NEG_IN	NEG_IN	
6	29	4																				POS_OUT
7	60	5	1	NEG_IN	NEG_IN	NEG_IN	INC_OUT	NEG_IN	INC_OUT	NEG_IN	NEG_IN	NEG_IN	INC_OUT	NEG_IN	NEG_IN	NEG_IN	NEG_IN	NEG_IN	NEG_OUT	NEG_IN	NEG_IN	
8	61	6		POS_OUT	NEG_IN	POS_OUT	NEG_IN	POS_OUT	INC_OUT	POS_OUT	NEG_IN	INC_OUT	NEG_IN	INC_OUT	INC_OUT	INC_OUT	NEG_OUT	INC_OUT	INC_OUT	POS_OUT	POS_OUT	
9	91	7																				POS_OUT
10	102	8		POS_OUT	INC_OUT	POS_OUT	POS_IN	POS_OUT	POS_IN	NEG_IN	POS_IN	NEG_IN	INC_OUT	NEG_IN	POS_IN	POS_OUT	POS_OUT	NEG_IN	POS_OUT	NEG_IN	NEG_IN	
11	109	9		POS_OUT	NEG_IN	POS_OUT	NEG_IN	POS_OUT	NEG_IN	NEG_IN	NEG_IN	INC_OUT	NEG_IN	POS_OUT	NEG_IN	POS_OUT	NEG_IN	POS_OUT	INC_OUT	INC_OUT	POS_IN	
12	117	10		NEG_IN	INC_OUT	NEG_OUT	INC_OUT	NEG_IN	NEG_OUT	NEG_IN	NEG_IN	NEG_IN	POS_IN	NEG_IN	NEG_OUT	NEG_IN	NEG_OUT	POS_IN	NEG_OUT	POS_IN	NEG_OUT	
13	139	11																				NEG_IN
14	143	12																				INC_OUT
15	159	13		NEG_IN	INC_OUT	NEG_IN	INC_OUT	NEG_IN	INC_OUT	POS_OUT	INC_OUT	POS_OUT	INC_OUT	POS_IN	NEG_OUT	NEG_IN	INC_OUT	NEG_OUT	INC_OUT	NEG_OUT	NEG_IN	
16	175	14																				NEG_OUT
17	177	15																				INC_OUT
18	185	16																				NEG_IN
19	195	17	3	NEG_IN	INC_OUT	NEG_IN	NEG_OUT	NEG_IN	NEG_OUT	POS_IN	POS_OUT	POS_IN	INC_OUT	POS_IN	INC_OUT	POS_IN	INC_OUT	NEG_IN	NEG_IN	NEG_OUT	NEG_IN	
20	209	18		NEG_IN	NEG_IN	NEG_IN	NEG_IN	NEG_IN	NEG_IN	NEG_IN	NEG_OUT	POS_IN	NEG_IN	POS_OUT	INC_OUT	NEG_IN	NEG_OUT	NEG_IN	NEG_IN	NEG_OUT	NEG_IN	
21	229	19	3	NEG_IN	NEG_IN	NEG_IN	INC_OUT	NEG_IN	INC_OUT	POS_IN	INC_OUT	INC_IN	INC_OUT	INC_IN	NEG_IN	NEG_IN	NEG_OUT	NEG_OUT	INC_OUT	NEG_IN	NEG_OUT	
22	244	20		POS_OUT	NEG_IN	NEG_IN	POS_IN	NEG_IN	NEG_IN	POS_OUT	NEG_IN	POS_OUT	NEG_IN	POS_OUT	NEG_IN	POS_OUT	NEG_IN	INC_OUT	INC_OUT	POS_OUT	POS_OUT	
23	245	21		POS_OUT	INC_OUT	NEG_IN	POS_IN	POS_OUT	INC_OUT	INC_IN	POS_IN	NEG_IN	INC_OUT	NEG_IN	NEG_OUT	NEG_IN	NEG_IN	NEG_IN	NEG_IN	INC_OUT	NEG_IN	
24	258	22	3	NEG_IN	INC_OUT	POS_OUT	NEG_OUT	NEG_IN	NEG_OUT	INC_OUT	INC_OUT	NEG_IN	NEG_IN	NEG_IN	INC_OUT	NEG_IN	INC_OUT	NEG_IN	NEG_IN	NEG_OUT	NEG_IN	
25	298	23	1	NEG_IN	NEG_IN	NEG_IN	NEG_IN	NEG_IN	NEG_IN	POS_IN	NEG_IN	POS_IN	NEG_IN	POS_IN	NEG_IN	POS_OUT	NEG_IN	POS_IN	POS_IN	POS_IN	POS_IN	
26	309	24																				INC_OUT
27	314	25																				NEG_IN
28	363	26		POS_OUT	NEG_IN	NEG_IN	NEG_IN	INC_OUT	NEG_IN	NEG_IN	NEG_IN	NEG_IN	NEG_IN	NEG_IN	NEG_OUT	POS_OUT	POS_OUT	INC_OUT	INC_OUT	NEG_IN	POS_IN	
29	366	27																				INC_OUT
30	402	28		POS_OUT	INC_OUT	POS_OUT	INC_OUT	POS_OUT	NEG_OUT	INC_OUT	POS_IN	NEG_OUT	INC_OUT	INC_OUT	NEG_OUT	POS_OUT	NEG_OUT	INC_OUT	INC_OUT	NEG_OUT	NEG_OUT	
31	405	29		POS_OUT	NEG_OUT	NEG_IN	NEG_IN	INC_OUT	INC_OUT	NEG_IN	NEG_IN	NEG_IN	NEG_IN	NEG_IN	NEG_IN	NEG_IN	NEG_OUT	INC_OUT	POS_OUT	NEG_OUT	NEG_OUT	
32	411	30	3	POS_IN	POS_IN	POS_IN	POS_OUT	POS_IN	POS_OUT	POS_OUT	INC_OUT	INC_OUT	NEG_OUT	NEG_OUT	INC_OUT	POS_IN	POS_IN	POS_IN	POS_OUT	POS_IN	POS_IN	
33	416	31	3	INC_OUT	INC_OUT	NEG_IN	INC_OUT	NEG_IN	NEG_OUT	NEG_IN	INC_OUT	NEG_IN	INC_OUT	NEG_IN	NEG_IN	NEG_IN	NEG_OUT	NEG_OUT	POS_OUT	NEG_IN	NEG_OUT	
34	431	32	3	INC_IN	INC_OUT	NEG_IN	INC_OUT	INC_IN	INC_OUT	NEG_IN	INC_OUT	NEG_IN	NEG_IN	NEG_IN	NEG_OUT	NEG_IN	INC_OUT	NEG_IN	NEG_IN	NEG_IN	NEG_IN	
35	448	33		NEG_IN	NEG_IN	INC_OUT	POS_OUT	NEG_IN	NEG_OUT	NEG_IN	NEG_IN	NEG_IN	POS_IN	NEG_IN	INC_OUT	INC_OUT	NEG_IN	POS_OUT	INC_OUT	NEG_OUT	POS_IN	
36	456	34																				
37	476	35		POS_OUT	INC_OUT	NEG_IN	NEG_IN	NEG_IN	INC_OUT	POS_OUT	NEG_IN	NEG_IN	INC_OUT	NEG_IN	NEG_OUT	NEG_IN	NEG_OUT	NEG_IN	NEG_IN	INC_OUT	NEG_IN	
38	494	36																				NEG_IN
39	499	37		POS_OUT	INC_OUT	NEG_IN	INC_OUT	NEG_IN	INC_OUT	INC_OUT	INC_OUT	NEG_IN	INC_OUT	NEG_IN	NEG_OUT	NEG_IN	INC_OUT	INC_OUT	INC_OUT	NEG_OUT	POS_OUT	
40	508	38																				NEG_IN
41	511	39	3	NEG_IN	INC_OUT	NEG_IN	NEG_OUT	NEG_IN	NEG_OUT	POS_IN	INC_OUT	POS_IN	INC_OUT	POS_IN	INC_OUT	INC_IN	INC_OUT	NEG_OUT	INC_OUT	INC_OUT	NEG_IN	
42	520	40		INC_OUT	NEG_IN	NEG_OUT	NEG_IN	INC_OUT	NEG_OUT	POS_OUT	NEG_OUT	NEG_IN	NEG_IN	NEG_IN	NEG_IN	INC_OUT	INC_OUT	POS_OUT	INC_OUT	POS_OUT	POS_IN	
43	525	41		NEG_IN	NEG_OUT	POS_OUT	NEG_OUT	POS_OUT	NEG_IN	NEG_IN	NEG_IN	NEG_IN	NEG_IN	NEG_IN	INC_OUT	NEG_IN	NEG_IN	INC_OUT	NEG_OUT	INC_OUT	NEG_OUT	
44	531	42		INC_OUT	INC_OUT	NEG_IN	NEG_IN	NEG_IN	NEG_IN	NEG_IN	NEG_IN	NEG_IN	NEG_IN	NEG_IN	NEG_IN	NEG_IN	INC_OUT	INC_OUT	NEG_IN	INC_OUT	POS_IN	
45	559	43	3	POS_IN	INC_OUT	POS_IN	NEG_IN	POS_IN	NEG_OUT	NEG_IN	INC_OUT	NEG_IN	NEG_OUT	NEG_IN	NEG_IN	INC_IN	NEG_OUT	INC_OUT	POS_IN	NEG_IN	INC_OUT	
46	565	44	1	NEG_IN	NEG_OUT	NEG_IN	NEG_IN	NEG_IN	INC_OUT	NEG_IN	NEG_OUT	NEG_IN	NEG_IN	NEG_IN	NEG_OUT	NEG_IN	INC_OUT	POS_OUT	POS_OUT	NEG_OUT	NEG_IN	
47	604	45	2	NEG_IN	NEG_OUT	NEG_IN	NEG_IN	NEG_IN	NEG_OUT	POS_OUT	NEG_IN	POS_IN	NEG_IN	INC_OUT	NEG_OUT	NEG_OUT	INC_OUT	INC_OUT	INC_OUT	POS_OUT	POS_OUT	
48	628	46																				NEG_IN
49	641	47		NEG_IN	NEG_OUT	POS_OUT	NEG_IN	POS_OUT	NEG_IN	NEG_IN	NEG_IN	NEG_IN	NEG_IN	NEG_IN	NEG_IN	NEG_IN	NEG_IN	INC_OUT	NEG_OUT	POS_OUT	NEG_OUT	
50	654	48	1	POS_OUT	INC_OUT	POS_OUT	NEG_IN	POS_OUT	NEG_IN	INC_OUT	NEG_IN	POS_IN	POS_IN	POS_IN	NEG_IN	POS_OUT	INC_OUT	NEG_OUT	INC_OUT	NEG_OUT	NEG_IN	
51	678	49		NEG_IN	INC_OUT	NEG_IN	NEG_IN	NEG_IN	INC_OUT	NEG_IN	NEG_IN	NEG_IN	INC_OUT	NEG_IN	NEG_OUT	NEG_IN	INC_OUT	NEG_IN	NEG_IN	NEG_OUT	NEG_IN	
52	695	50	3																			

Figure S1 illustrates a detailed summary of the data processing phase.

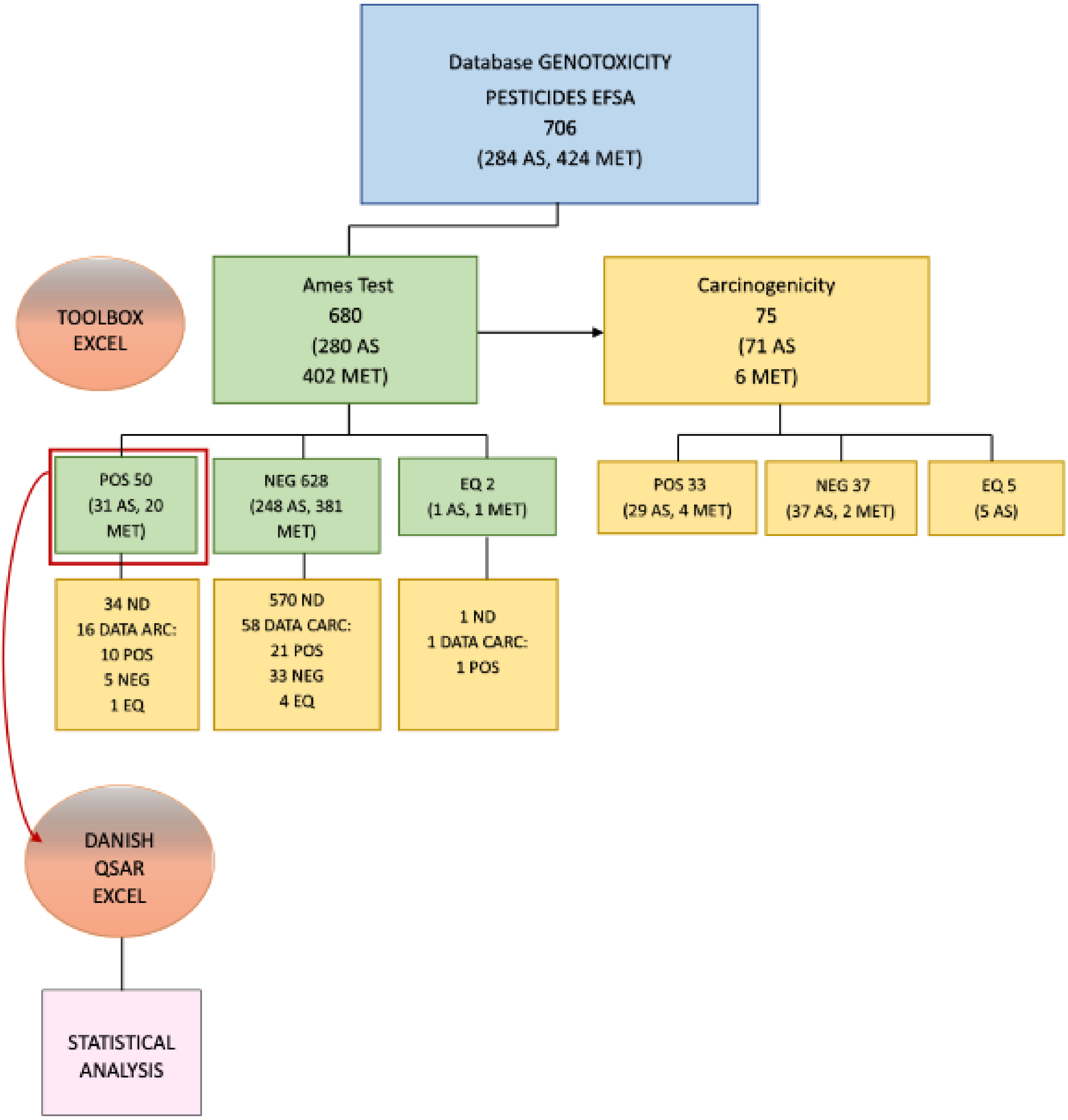

Figure S1. A comprehensive summary of the steps performed on the EFSA Genotoxicity database. It includes counts and classifications of active substances (ASs) and metabolites (METs), along with the application results of the Ames test and experimental carcinogenicity data, categorized as **POS** (positive), **NEG** (negative), **EQ** (equivocal), and **ND** (no data available). The data are visually represented using a color-coded schema: **Green**: genotoxicity data; **Yellow**: carcinogenicity data; **Pink**: statistical analysis; **Orange**: software used in different steps; and **Blue**: the source database and any modifications applied. It is important to note that some substances appear both as active substances and metabolites, but they are counted individually in the overall totals.
